# IgA Subclasses and Free Light Chains in Celiac Disease: A Pilot Study

**DOI:** 10.3390/ijms27104589

**Published:** 2026-05-20

**Authors:** Valeria Carnazzo, Viviana Grieco, Valerio Basile, Serena Redi, Mariapaola Marino, Gabriele Ciasca, Francesco Bondanini, Umberto Basile

**Affiliations:** 1 Department of Clinical Pathology, Santa Maria Goretti Hospital, 04100 Latina, Italy; v.carnazzo@ausl.latina.it (V.C.); s.redi@ausl.latina.it (S.R.); u.basile@ausl.latina.it (U.B.); 2 Unità Operativa Complessa Laboratorio Analisi, Ospedale Sant’Eugenio ASL RM 2, 00144 Rome, Italy; viviana.grieco@aslroma2.it (V.G.); francesco.bondanini@aslroma2.it (F.B.); 3 Clinical Pathology Unit and Cancer Biobank, Department of Research and Advanced Technologies, Regina Elena National Cancer Institute I.R.C.C.S., 00144 Rome, Italy; valeriobasile90@gmail.com; 4 Dipartimento di Medicina e Chirurgia Traslazionale, Sezione di Patologia Generale, Università Cattolica del Sacro Cuore, 00168 Rome, Italy; 5 Fondazione Policlinico Universitario “A. Gemelli” I.R.C.C.S., 00168 Rome, Italy; 6 Dipartimento di Neuroscienze, Sezione di Fisica, Università Cattolica del Sacro Cuore, 00168 Rome, Italy

**Keywords:** celiac disease, biomarkers, free light chains, IgA subclasses, autoantibodies

## Abstract

Celiac disease (CD) is an autoimmune enteropathy of the small intestine affecting genetically susceptible individuals, characterized by an aberrant immune response to gliadin and sustained IgA-driven inflammation. IgA exists in two main subclasses, IgA1 and IgA2, which differ in distribution and function, but their profile in CD remains poorly characterized. Circulating free light chains (FLCs) are markers of B-cell activation and immune dysregulation, yet their role in CD has not been fully explored. The aim of this study was to characterize IgA subclasses and FLC profiles in newly diagnosed celiac patients. We analyzed sera from 108 CD patients and 29 healthy controls, assessing conventional serological markers (anti-tissue transglutaminase and anti-endomysial antibodies), together with total IgA, IgA1, IgA2, and FLC levels using a turbidimetric method. CD patients exhibited higher total IgA levels and an increased IgA1/IgA2 ratio, alongside a decreased k/λ ratio; these differences remained significant after adjustment for age and sex. When combined in a multivariable logistic model, these biomarkers yielded an AUC of 0.827, suggesting that the parameters identified in the univariate analyses provide complementary, non-redundant information that jointly highlights a reorganization of the humoral immune response. Due to the limited sample size, our results need confirmation in larger cohorts. However, our findings suggest a reorganization of the IgA compartment in CD, with selective expansion of IgA1 and preferential λ light chain usage, highlighting coordinated alterations in the humoral immune response. The integration of such markers, potentially in combination with -omics approaches, may contribute to a more refined and less invasive characterization of celiac disease.

## 1. Introduction

Celiac disease (CD) is a chronic autoimmune enteropathy of the small intestine triggered by the ingestion of gluten in genetically susceptible individuals carrying HLA-DQ2 or HLA-DQ8 alleles [1,2]. CD affects approximately 1% of the global population, with underdiagnosis in many cases [3,4,5]. A complete understanding of the pathogenesis is lacking, and other environmental factors are likely to play a role by altering the balance between inflammation and tolerance. The disease is characterized by an aberrant immune response to gluten peptides, primarily gliadin. Upon ingestion, gliadin peptides resist complete digestion and cross the intestinal epithelium, where they are deamidated by tissue transglutaminase (t-TG) [6]. Deamidated gliadin binds more efficiently to HLA-DQ2 or HLA-DQ8 molecules on antigen-presenting cells, leading to the activation of CD4+ T cells in the lamina propria. These activated T cells release pro-inflammatory cytokines, IFN-y and TNF-α, which polarize an adaptive immune response resulting in the disruption of epithelial integrity and recruitment of additional immune cells [7,8]. Similar cytokine-mediated inflammatory cascades involving IL-6 signaling have been described as key amplifiers of immune-mediated tissue inflammation [9]. The combined effect of cellular and humoral immune responses results in villous atrophy, crypt hyperplasia, and increased intraepithelial lymphocytes, ultimately impairing nutrient absorption. Clinically, immune dysregulation is paralleled by a broad spectrum of gastrointestinal symptoms, including diarrhea, bloating, abdominal pain, and weight loss [10,11]. Moreover, extraintestinal complications may arise, such as anemia, osteoporosis, neurological disturbances, and increased risk of autoimmune comorbidities, reflecting the systemic impact of chronic intestinal inflammation [12,13,14,15].

Immunoglobulin A (IgA) is the primary isotype in gut mucosal secretion, protecting the intestinal epithelium from commensal bacteria, dietary antigens, and pathogenic microbes [16]. Different cytokines and soluble factors, such as IL-6, TGF-β, APRIL, and BAFF, initially produced by innate immune cells, but also by epithelial cells, can drive distinct CD40-dependent and CD40-independent (T-independent) immunoglobulin heavy chain α class switching in mucosal-associated lymphoid tissue (MALT). Although less abundant in serum than IgG, IgA isotype antibodies are considered a more reliable biomarker in an enteropathy such as CD, because the autoimmune response is primarily mucosal; even if systemic and mucosal IgA responses are compartmentalized, serum IgA autoantibodies reflect mucosal immune activation and represent sensitive and specific markers of the gut immune response [17].

The immune dysregulation at the basis of CD involves B-cell activation and the release of disease-specific autoantibodies, such as anti-tissue transglutaminase (anti-tTG) and anti-endomysial (EMA) antibodies, as well as anti-deamidated gliadin peptides [18,19,20,21]. Among these, the assessment of anti-tTG antibodies of the IgA isotype is preferred for clinical use, because it shows good sensitivity, greater than 90%, and a high specificity of around 95% [22].

IgA exists in two main subclasses, IgA1 and IgA2, which differ in distribution and function [23,24]. IgA1 is the major subclass and accounts for 80% of total IgA in serum, whereas IgA2 is the major subclass in secretions such as milk. Alterations in total IgA levels and subclass distribution may provide insight into disease mechanisms and immune dysregulation occurring in CD.

Immunoglobulin free light chains (FLC) kappa (k) and lambda (λ) are produced in slight excess over heavy chains during the synthesis of intact Ig by plasma cells, showing a short half-life (2–6 h), and undergoing renal excretion in normal conditions [25]; therefore, in subjects with normal kidney function, an increase in their serum levels is considered a marker of immunoglobulin production or underlying immunological abnormalities [26,27]. While the clinical significance of FLC has been extensively studied in plasma cell disorders and autoimmune diseases [28,29], its role in celiac disease remains poorly characterized.

In this study, we aimed to delineate the immunological profiles of IgA subclasses and FLC in patients with CD and explore their potential associations. CD diagnosis is based on mucosal damage in small intestinal biopsies in patients with circulating disease-specific antibodies but may remain underdiagnosed [3,4,5]. We retain that the evaluation of IgA subclasses and serum FLC in patients may improve our understanding of the humoral immune response and potentially identify novel biomarkers for disease activity and severity, thereby improving the management of initial diagnosis.

## 2. Results

### 2.1. Demographic and Immunological Characteristics

We enrolled 29 controls and 108 patients with newly diagnosed celiac disease. All patients were positive for IgA anti-tTG and EMA antibodies (abs), and the diagnosis of celiac disease was confirmed by duodenal biopsy. We compared groups using an immunologic panel comprising serum FLCs with their k/λ ratio, total IgA and subclasses (IgA1 and IgA2), anti-tTG IgA, and basic demographics (Table 1).

In the CD cohort, 64% of patients were female, and 36% were male. No significant difference in sex distribution was observed between patients and controls (62% vs. 64%, *p* > 0.9), nor in age (25 [20–34] vs. 27 [22–45] years, *p* = 0.15). As expected, anti-tTG IgA Abs were detectable only in the celiac group (median 212 U/mL, IQR 37–1141) and were absent in controls. In univariate comparisons, patients showed higher total IgA (216 [155–304] vs. 178 [114–237] mg/dL, *p* = 0.006), higher IgA1 (*p* = 0.006), and a higher IgA1/IgA2 ratio (4.72 [3.33–6.66] vs. 3.70 [2.69–5.06], *p* = 0.009). The k/λ ratio was lower in patients than in controls (1.02 [0.80–1.23] vs. 1.22 [1.01–1.51], *p* = 0.004), whereas k, λ, and IgA2 did not differ significantly. Notably, median k/λ ratios in both groups remained within the commonly used reference interval (0.26–1.65), although the difference was clearly statistically significant; median total IgA and subclass values also fell within typical adult reference intervals.

The age- and sex-adjusted analysis (grey panel in Table 1) confirmed the univariate findings for the k/λ ratio, total IgA, and the IgA1/IgA2 ratio. With controls as the reference, the adjusted difference (patients − controls) was −0.22 for the k/λ ratio (95% CI −0.40 to −0.04; *p* = 0.021), +1.3 for the IgA1/IgA2 ratio (95% CI 0.36 to 2.3; *p* = 0.008), and +78 mg/dL for total IgA (95% CI 1.3 to 155; *p* = 0.046). Other markers were not significant after adjustment.

**Figure 1:**
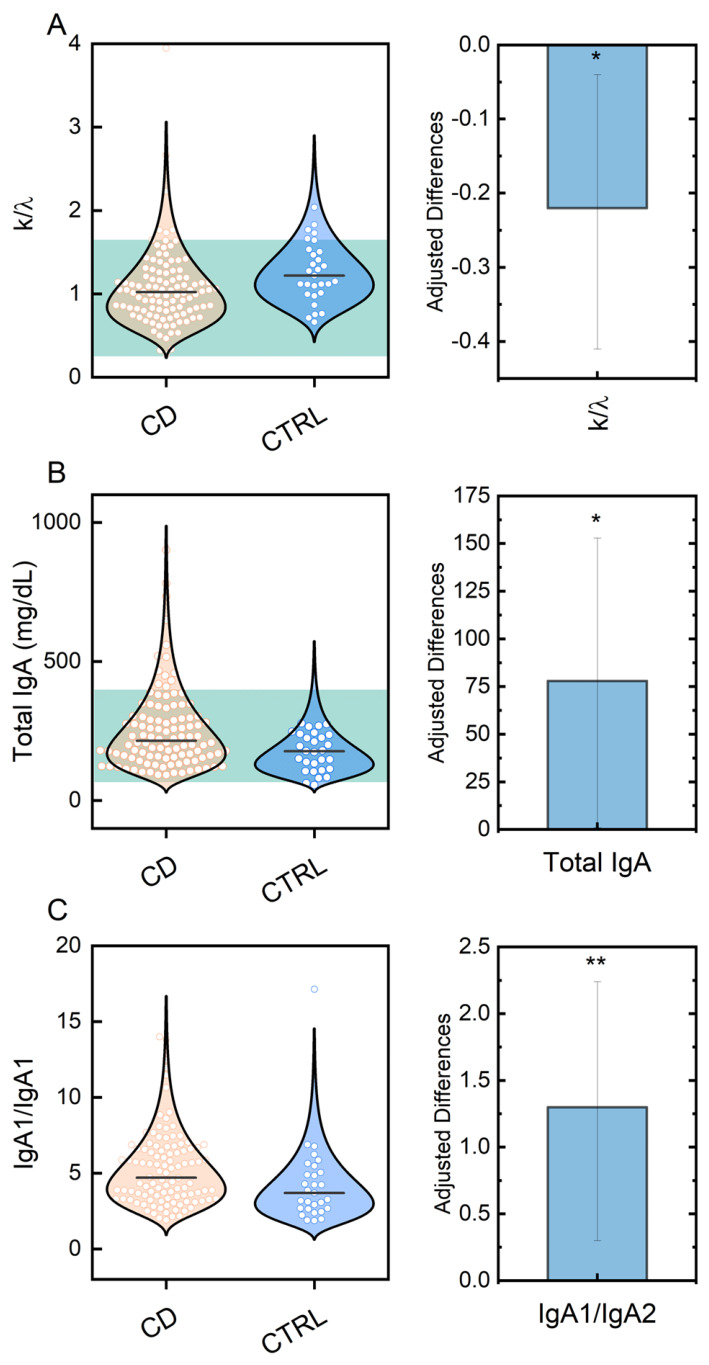
Distributions and adjusted between-group differences for key immunologic markers in celiac patients and controls. (**A**) k/λ ratio (unitless); (**B**) total IgA (mg/dL); (**C**) IgA1/IgA2 ratio. Left panels: violin plots with individual observations (dots) and group medians (horizontal bars). Teal shaded areas indicate commonly used reference intervals for k/λ and total IgA (panels (**A**,**B**)). Right panels (blue boxes): age- and sex-adjusted differences (celiac − control) with 95% confidence intervals derived from linear models. Asterisks denote statistical significance (* *p* < 0.05, ** *p* < 0.01).

Figure 1 (left panels) presents violin plots of the variables that were significant both in the univariate and in the age- and sex-adjusted analyses—namely the k/λ ratio (Figure 1A), total IgA (Figure 1B), and the IgA1/IgA2 ratio (Figure 1C)—illustrating their distribution in controls versus patients. Figure 1 (right panels) shows the corresponding adjusted differences, together with 95% CIs, and significance levels denoted by asterisks.

### 2.2. Integrated Analysis of Circulating Immunological Biomarkers

**Figure 2:**
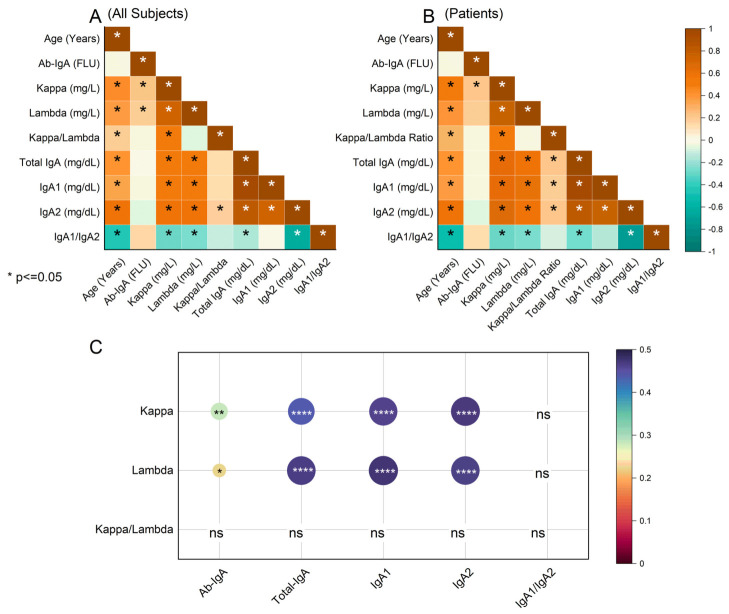
Correlation analysis of immunologic variables. (**A**) Spearman correlation matrix across all enrolled subjects. (**B**) Spearman correlation matrix within celiac patients only. Tiles are colored by Spearman’s ρ (−1 to +1); asterisks (*) denote *p* < 0.05. Variables: Age (years), IgA antibodies (Ab-IgA FLU), kappa (mg/L), lambda (mg/L), kappa/lambda ratio, total IgA (mg/dL), IgA1 (mg/dL), IgA2 (mg/dL), IgA1/IgA2. (**C**) Age- and sex-adjusted partial Spearman correlations for kappa, lambda, and the kappa/lambda ratio versus Ab-IgA, total IgA, IgA1, IgA2, and IgA1/IgA2; circle size reflects |ρ|; color scale indicates *p*-value; asterisks/”ns” annotate significance (* *p* < 0.05, ** *p* < 0.01, **** *p* < 0.0001; “ns” indicates not significant); FLU = Fluorescence Units.

**Figure 3:**
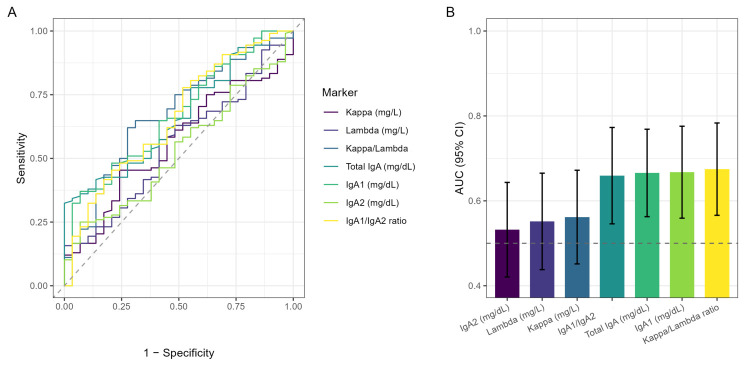
Classification performance of circulating markers. (**A**) ROC curves for single markers—kappa (mg/L), lambda (mg/L), kappa/lambda ratio, total IgA (mg/dL), IgA1 (mg/dL), IgA2 (mg/dL), and IgA1/IgA2 ratio—contrasting celiac patients with controls; the diagonal dashed line indicates a random classifier. (**B**) bar plot of the corresponding AUCs with 95% confidence intervals for each marker; the horizontal dashed line marks AUC = 0.5.

In Figure 2, we show the correlation structure of the immunologic markers. Figure 2A displays the Spearman correlation matrix across all enrolled subjects (asterisks denote *p* < 0.05). Most immunologic readouts increase with age, consistent with an age-related drift in humoral immunity [30,31]. Here, the IgA1/IgA2 ratio seems to be an exception, likely reflecting a stronger age association of IgA2 than IgA1. Laboratory immunologic variables are positively intercorrelated, indicating a coordinated activation across total IgA, IgA subclasses, and free light chains, as observed in other papers [1,2,3,4]. Notably, anti-tTG IgA abs show only weak or absent correlations with these global markers, suggesting complementary—rather than redundant—information that could be combined in circulating signatures, as investigated in Figure 3.

Figure 2B repeats the analysis within celiac patients only; the correlation profile remains essentially unchanged, suggesting that these couplings are independent of disease status in our clinical setting. The loss of significance for the λ—anti-tTG IgA pair is plausibly due to the combination of a small effect size and reduced sample size.

Figure 2C displays age- and sex-adjusted (partial Spearman) correlations focusing on k and λ versus total IgA, IgA1, IgA2, and anti-tTG IgA abs. After adjustment, k correlated with anti-tTG IgA (ρ = 0.282; 95% CI 0.097–0.449; *p* = 0.003), total IgA (ρ = 0.437; 0.289–0.565; *p* = 1.32 × 10^−7^), IgA1 (ρ = 0.460; 0.315–0.584; *p* = 2.19 × 10^−8^), and IgA2 (ρ = 0.471; 0.328–0.594; *p* = 8.93 × 10^−9^). Likewise, λ correlated with anti-tTG IgA abs (ρ = 0.223; 0.034–0.397; *p* = 0.0215), total IgA (ρ = 0.466; 0.322–0.589; *p* = 1.42 × 10^−8^), IgA1 (ρ = 0.476; 0.333–0.597; *p* = 6.38 × 10^−9^), and IgA2 (ρ = 0.464; 0.319–0.587; *p* = 1.69 × 10^−8^). The k/λ and IgA1/IgA2 ratios did not show significant partial correlations—either with each other or with the single-analyte measures—but did differ between patients and controls (see Table 1).

### 2.3. Investigation of the Discriminatory Ability of Individual Markers

To further support the presence of a reorganization of the humoral immune response, we evaluated the discriminatory ability of individual markers in distinguishing celiac patients from controls. Figure 3A shows the ROC curves for all investigated parameters, while Figure 3B reports the corresponding AUCs with 95% confidence intervals.

Markers showing AUC values above 0.5 included the k/λ ratio (AUC = 0.68; 95% CI 0.57–0.78; *p* = 0.004), IgA1 (0.67; 0.56–0.78; *p* = 0.006), total IgA (0.67; 0.56–0.77; *p* = 0.006), and the IgA1/IgA2 ratio (0.66; 0.55–0.77; *p* = 0.009). In contrast, k and λ light chains and IgA2 showed AUC values close to chance level, reflecting minimal individual contribution.

Overall, these results indicate that individual markers have limited discriminatory ability when considered in isolation and are not suitable as standalone diagnostic tools. However, their consistent behavior reinforces the immunological reorganization observed in Figure 1 and Table 1, supporting the presence of a coordinated shift in the IgA-related immune response in celiac disease.

### 2.4. Evaluation of the Integrative Information of Circulating Biomarkers

In Table 2 we reported the ROC AUC values for each individual biomarker. To explore whether the investigated parameters provide complementary information, we fitted a multivariable logistic regression model including total IgA, IgA1, the IgA1/IgA2 ratio, and the k/λ ratio.

Model selection based on AIC and BIC criteria retained all variables, suggesting that each marker contributes non-redundant information (Table 3). In the final model, higher total IgA and a higher IgA1/IgA2 ratio were associated with increased odds of celiac disease, whereas a higher kλ ratio was associated with reduced odds; IgA1 showed a smaller contribution.

**Figure 4:**
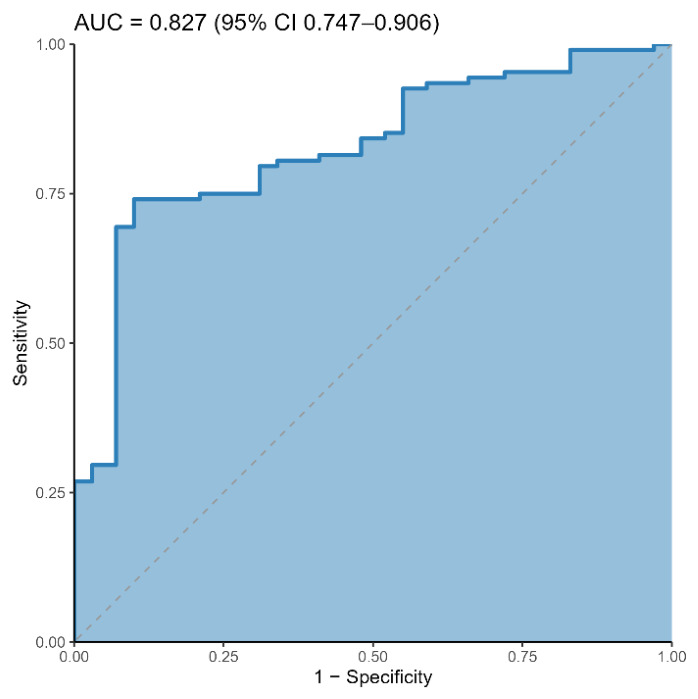
Receiver operating characteristic (ROC) curve for the multivariable logistic regression combining total IgA (mg/dL), IgA1 (mg/dL), IgA1/IgA2 ratio, and k/λ ratio (outcome: celiac = 1, CTRL = 0). The ROC is shown as a step function with the shaded area under the curve; the diagonal dashed line indicates chance level (AUC = 0.5). AUC and its 95% CI are reported on the plot.

The combined model achieved an AUC of 0.827 (95% CI 0.747–0.906), indicating improved discrimination compared to individual markers (Figure 4). Overall, these results support the presence of complementary contributions across markers, consistent with the immunological reorganization observed in Figure 1 and Table 1 [32].

## 3. Discussion

The present study aimed to characterize the distribution of IgA subclasses and free light chains (FLCs) in celiac disease, with the goal of gaining further insight into the organization of the humoral immune response in this condition.

Current serological markers, such as anti-tTG and EMA antibodies, remain central for diagnosis and follow-up, although analytical variability and borderline results may still occur in specific contexts, including uncertainty in the definition of cut-off values and differences in calibration procedures for anti-tTG IgA assays [33].

In this framework, the identification of additional circulating features may contribute to a more comprehensive description of disease-associated immune alterations, rather than serving as stand-alone diagnostic tools.

The biological immunomodulation of IgA and their regulation factors are still insufficiently characterized. Increasing evidence suggests that IgA acts in many immune responses as a regulator not only in gut homeostasis, highlighting its controversial role with both pro- and anti-inflammatory effects [23]. Local inflammation may drive immune polarization in response to different stimuli; in this scenario, the balance between TGF-β and proinflammatory cytokines seems to play a key role, as also previously described in other settings [17,34,35,36]. Understanding these dynamics could help explain patient-specific variations in humoral responses, paving the way for the development of personalized therapeutic strategies.

CD patients exhibited higher total IgA levels and an increased IgA1/IgA2 ratio, alongside a decreased k/λ ratio, with these findings persisting after adjustment for age and sex (Table 1). These alterations reflect polyclonal activation of the IgA compartment and reorganization of the antibody response, within a broader context of mucosal immune dysregulation, as observed in other clinical settings such as nephropathy [37]. From a pathophysiological perspective, the selective increase in IgA1 likely results from chronic intestinal inflammation driven by continuous antigenic stimulation from gliadin and other gluten components, leading to the expansion of IgA1-producing plasma cells in both mucosal and systemic compartments [38]. This observation supports the concept that chronic antigen exposure can imprint systemic immune alterations, not limited to the gut mucosa.

Concurrently, the reduced k/λ ratio suggests altered clonal selection of B cells or preferential recruitment of plasma cells producing λ chains, a phenomenon observed in other mucosal autoimmune conditions [39]. Our result is consistent with an original observation reported by Faure et al. [40] describing an increased number of plasma cells producing λ chains in samples from mucosal tissues, another feature reflecting the autonomy of MALT in humans. In addition, we previously reported, in another autoimmune setting, that a different trend between k and λ is specifically correlated with two different subgroups of affected patients, driving different responses to the autoantigen [41]. These data suggest that light chain usage may reflect differences in underlying immune responses and could be explored as a potential stratification feature.

Collectively, these changes reflect not only a gluten-specific humoral response but also a broader dysregulation of the IgA-mediated immune compartment in CD patients compared with healthy individuals.

Interestingly, the k/λ and IgA1/IgA2 ratios did not show significant partial correlations—either with each other or with the single-analyte measures (Figure 3)—while still exhibiting between-group differences (Table 1). This pattern further suggests that the ratios might carry information that is complementary to absolute concentrations, which could be advantageous when building combined circulating signatures. This point is particularly relevant in pediatrics, where the European Society of Pediatric Gastroenterology Hepatology and Nutrition (ESPGHAN) criteria permit confirmation of celiac disease without duodenal biopsy in selected cases with very high tTG-IgA and confirmatory EMA abs [42]. Moreover, these ratios may reflect subtle immune imbalances that are not captured by single-analyte measurements, emphasizing their potential role in risk stratification and monitoring. Additional circulating markers could therefore be clinically useful in such biopsy-sparing contexts.

When combined in a multivariable model, these parameters showed improved discriminative performance compared to individual analytes (AUC = 0.827; Table 2, Figure 4), supporting the notion that they capture complementary aspects of the humoral immune response. Rather than indicating immediate clinical applicability, this result suggests that integrated immunological profiles may provide a more informative representation of disease-associated alterations. From this perspective, such combined signatures may serve as a framework for future investigations aimed at refining disease characterization, particularly in contexts where a more nuanced assessment of immune activation is required.

These insights deepen our understanding of disease-specific humoral dysregulation and suggest that circulating immune profiles may complement conventional serology in the characterization of celiac disease.

## 4. Materials and Methods

### 4.1. Patients and Study Design

The study included 137 subjects, divided into two groups: 108 newly diagnosed CD patients and 29 healthy controls (CTRL). CD diagnosis was established at Fondazione Policlinico A. Gemelli-I.R.C.C.S. (Rome) during screening investigations, based on the presence of anti–tissue transglutaminase (tTG-IgA) and anti-endomysial (EMA) antibodies together with duodenal biopsy findings consistent with celiac disease; all patients were in a silent-asymptomatic state of CD according to Tonutti E et al. [43], consisting of the presence of positive CD-specific antibodies, HLA typing positive for specific alleles, and small-bowel biopsy findings compatible with CD but without sufficient symptoms and signs to warrant clinical suspicion of CD.

Serum samples were collected at the time of diagnosis from patients prior to initiation of a gluten-free diet. Sera were stored at −20 °C and subsequently transported to another clinical center (S. M. Goretti Hospital in Latina) for the evaluation of total IgA, IgA1, IgA2, the IgA1/IgA2 ratio, k and λ FLCs, and the k/λ ratio. Total IgA levels were checked before serology: none of the selected patients had selective IgA deficiency. Subjects with other active autoimmune diseases, ongoing infections, or immunosuppressive therapies were excluded. The study was conducted in accordance with the Declaration of Helsinki and was approved by the Ethics Committee of Università Cattolica del Sacro Cuore (Immuno-HVR ID: 2080). All participants provided written informed consent.

### 4.2. Conventional Biomarkers: Total IgA and Autoantibodies Assessment

Total serum IgA levels were measured using a fully automated chemiluminescent immunoturbidimetric method. This approach quantifies the increasing formation of insoluble immune complexes when IgA in serum binds to a specific anti-IgA antibody. The procedure includes several steps: initially, the sample is incubated with a stabilizing buffer. A baseline measurement (“blank value”) is taken, followed by the addition of the anti-IgA antibody. The formation of antigen-antibody complexes increases turbidity, which is proportional to IgA concentration and measured by the instrument (ARCHITECT i2000 immunoassay analyzer—Abbott Laboratories, Abbott Park, IL, USA). In adults, total IgA normal levels are typically between 60 and 400 mg/dL.

Anti-tTG IgA abs were measured using a next-generation multi-parametric PMAT (Particle Multi-Analyte Technology) platform (APTIVA, Inova Diagnostics, Inc., a Werfen company; headquartered in San Diego, CA, USA). This fluorometric technique involves incubating the sample with antigen-coated microspheres, washing, and adding a fluorescently labeled secondary antibody. Paramagnetic particles are aligned and read optically using a dual-LED source, with signals captured by a high-resolution CCD sensor and compared with analyte-specific calibration curves, ensuring accuracy and reproducibility.

EMA IgA abs were determined by indirect immunofluorescence on primate liver sections (EUROIMMUN FA 1913-1005 A) using the TITERPLANE technique. Serum samples diluted 1:10 were incubated on BIOCHIP slides, washed with PBS-Tween buffer, treated with fluorescein-labeled anti-IgA conjugate, and examined under a fluorescence microscope (EUROPattern, 450–490 nm). The reference negative titer was <1:10 for IgA (EUROIMMUN Medizinische Labordiagnostika AG, Lübeck, Germany).

Results of IgA antibodies were expressed in Fluorescence Units (FLU).

### 4.3. Measurement of Investigated Biomarkers: Free Light Chains (k and λ) and IgA Subclasses

Free k and λ chains of immunoglobulins were quantified using an Optilite turbidimeter (Binding Site Group Ltd., Birmingham, UK) based on turbidimetric principles. The determination of soluble antigen concentration by turbidimetric methods involves the reaction with specific polyclonal sheep antiserum to form insoluble complexes. When light passes through the suspension formed, a portion of the light is transmitted and focused onto a photodiode by an optical lens system. The decrease in transmitted light intensity reflects the formation of antigen–antibody complexes, providing reliable quantitative measurements. Serum samples for k chains were pre-diluted 1:10 and calibrated against a 7-point standard curve (2.9–127.0 mg/L). For λ chains, the dilution was 1:8 with a 5.2–139.0 mg/L calibration range. Measurements below the lower limit were adjusted for enhanced sensitivity (0.6 mg/L for k, 1.3 mg/L for λ), while samples exceeding the upper limit were automatically diluted to remain within the linear range. The instrument also corrects for the prozone effect caused by antigen excess.

The same turbidimetric method was applied to quantify IgA1 and IgA2 subclasses using dedicated calibration curves (Optilite, Binding Site Group Ltd., Birmingham, UK). Specific polyclonal sheep anti-IgA1 or anti-IgA2 antibodies bind to the IgA in the sample, forming insoluble complexes. Assays are optimized for sensitivity to identify low levels of IgA subclasses in children or in patients with deficiencies. Normal reference ranges for IgA subclasses are highly age-, method-, and sample type-dependent; in our laboratory, IgA1: 76–328 mg/dL, IgA2: 6.9–114.3 mg/dL. The lower limit of the IgA1 measurement range is 35 mg/L, whereas that for IgA2 is 5.0 mg/L.

### 4.4. Statistical Analysis

Data visualization was performed using OriginPro 2022 and R (version 4.9.1, R Foundation for Statistical Computing). Summary statistics are reported as median and interquartile range (IQR) for non-normally distributed variables or mean and standard deviation (SD) for approximately Gaussian variables, as assessed by distributional inspection. Group comparisons were conducted with Fisher’s exact test for categorical data and Wilcoxon rank-sum or Student’s *t*-test for continuous data, depending on distributional assumptions. Tables were generated with the *gtsummary* package in R.

Bivariate correlations were evaluated with Spearman’s rank correlation, displayed as correlation matrices. To account for age and sex as potential confounders, partial Spearman correlations were estimated in R using the ppcor framework, with confidence intervals obtained by bootstrap resampling.

Diagnostic performance of each marker was assessed with receiver operating characteristic (ROC) analysis and the area under the curve (AUC) with 95% confidence intervals [32]. Markers were first evaluated individually and then combined in a multivariable logistic regression model (generalized linear model with binomial link). According to previous papers investigating similar variables [44], candidate predictors were selected from those showing significant univariable AUCs. Model selection was guided by stepwise procedures using both the Akaike information criterion (AIC) and the Bayesian information criterion (BIC), which converged on the same final specification. The multivariable classifier was evaluated by ROC curve analysis.

## 5. Conclusions

CD diagnosis relies on serology and duodenal biopsy, with biopsy-sparing criteria applicable in selected pediatric cases [45,46]. Challenges remain, including assay variability, seronegative or borderline cases, and the need for non-invasive monitoring of gluten-free diet adherence.

In this study, circulating markers such as total IgA, the IgA1/IgA2 ratio, and the k/λ ratio showed consistent differences between celiac patients and controls. The increase in total IgA and in the IgA1/IgA2 ratio is consistent with sustained humoral activation, while the reduction in the k/λ ratio suggests a preferential recruitment of plasma cells producing λ light chains. When considered jointly, these parameters provide complementary, non-redundant information suggesting a complex reorganization of the humoral immune response.

Although limited by the sample size and study design, these results provide additional insight into immune alterations in celiac disease and may serve as a basis for further studies aimed at integrating such profiles with established clinical parameters. Validation in larger and independent cohorts will be required to better define their potential diagnostic relevance.

## Figures and Tables

**Table 1:** Demographic and serological correlates between CD and controls.

Variable	CTRL N = 29 ^1^	CD N = 108 ^1^	*p*-Value ^2^	Adjusted Differences ^3^	95% CI ^3^	*p*-Value ^3^
**Sex**			>0.9			
▪ F	18 (62%)	69 (64%)				
▪ M	11 (38%)	39 (36%)				
**Age (years)**	25 (20, 34)	27 (22, 45)	0.15			
**anti-tTG IgA (U/mL)**	NA	212 (37, 1141)				
**Free k (mg/L)**	20 (15, 25)	17 (12, 23)	0.3	−0.40	−7.8, 7.0	>0.9
**Free λ (mg/L)**	14 (12, 20)	17 (12, 21)	0.4	2.7	−4.0, 9.3	0.4
**k/λ ratio**	1.22 (1.01, 1.51)	1.02 (0.80, 1.23)	0.004	−0.22	−0.40, −0.04	0.021
**Total IgA (mg/dL)**	178 (114, 237)	216 (155, 304)	0.006	78	1.3, 155	0.046
**IgA1 (mg/dL)**	147 (90, 172)	171 (123, 249)	0.006	32	−22, 87	0.2
**IgA2 (mg/dL)**	34 (20, 59)	38 (24, 70)	0.6	1.6	−10, 13	0.8
**IgA1/IgA2**	3.70 (2.69, 5.06)	4.72 (3.33, 6.66)	0.009	1.3	0.36, 2.30	0.008

^1^ n (%); Median (Q1, Q3); ^2^ Fisher’s exact test; Wilcoxon rank sum test; ^3^ ANCOVA; Abbreviation: CI = Confidence Interval; gray panel indicates age- and sex-adjusted analysis.

**Table 2:** ROC AUC with 95% CI and *p*-values.

Marker	AUC (95% CI)	*p*-Value
k/λ	0.68 (0.57–0.78)	0.004
IgA1 (mg/dL)	0.67 (0.56–0.78)	0.006
Total IgA (mg/dL)	0.67 (0.56–0.78)	0.006
IgA1/IgA2	0.66 (0.55–0.77)	0.009
Free k (mg/L)	0.66 (0.55–0.77)	0.309
Free λ (mg/L)	0.56 (0.45–0.67)	0.396
IgA2 (mg/dL)	0.53 (0.42–0.64)	0.598

**Table 3:** Multivariable logistic regression combining circulating markers.

Variable	β (SE)	OR (95% CI)	*p*-Value
Total IgA (mg/dL)	0.020 (0.006)	1.02 (1.01–1.04)	<0.001
k/λ	−1.16 (0.52)	0.31 (0.11–0.88)	0.025
IgA1/IgA2	0.48 (0.16)	1.62 (1.22–2.26)	0.002
IgA1 (mg/dL)	−0.010 (0.005)	0.99 (0.98–1.00)	0.048

(outcome: celiac = 1, CTRL = 0). Coefficients are on the log-odds scale; OR with 95% CI are exponentiated effects.

## Data Availability

The data presented in this study are available upon reasonable request to the corresponding authors.
